# DNA strand asymmetry generated by CpG hemimethylation has opposing effects on CTCF binding

**DOI:** 10.1093/nar/gkad293

**Published:** 2023-04-24

**Authors:** Stacey L Thomas, Ting-Hai Xu, Brittany L Carpenter, Steven E Pierce, Bradley M Dickson, Minmin Liu, Gangning Liang, Peter A Jones

**Affiliations:** Department of Epigenetics, Van Andel Institute, Grand Rapids, MI 49503, USA; Department of Epigenetics, Van Andel Institute, Grand Rapids, MI 49503, USA; Department of Structural Biology, Van Andel Institute, Grand Rapids, MI 49503, USA; Department of Epigenetics, Van Andel Institute, Grand Rapids, MI 49503, USA; Department of Neurodegenerative Science, Van Andel Institute, Grand Rapids, MI 49503, USA; Department of Epigenetics, Van Andel Institute, Grand Rapids, MI 49503, USA; Department of Epigenetics, Van Andel Institute, Grand Rapids, MI 49503, USA; Department of Urology, Keck School of Medicine, University of Southern California, Los Angeles, CA 90089, USA; Department of Epigenetics, Van Andel Institute, Grand Rapids, MI 49503, USA

## Abstract

CpG methylation generally occurs on both DNA strands and is essential for mammalian development and differentiation. Until recently, hemimethylation, in which only one strand is methylated, was considered to be simply a transitory state generated during DNA synthesis. The discovery that a subset of CCCTC-binding factor (CTCF) binding sites is heritably hemimethylated suggests that hemimethylation might have an unknown biological function. Here we show that the binding of CTCF is profoundly altered by which DNA strand is methylated and by the specific CTCF binding motif. CpG methylation on the motif strand can inhibit CTCF binding by up to 7-fold, whereas methylation on the opposite strand can stimulate binding by up to 4-fold. Thus, hemimethylation can alter binding by up to 28-fold in a strand-specific manner. The mechanism for sensing methylation on the opposite strand requires two critical residues, V454 and S364, within CTCF zinc fingers 7 and 4. Similar to methylation, CpG hydroxymethylation on the motif strand can inhibit CTCF binding by up to 4-fold. However, hydroxymethylation on the opposite strand removes the stimulatory effect. Strand-specific methylation states may therefore provide a mechanism to explain the transient and dynamic nature of CTCF-mediated chromatin interactions.

## Graphical Abstract

**Graphical Abstract:**
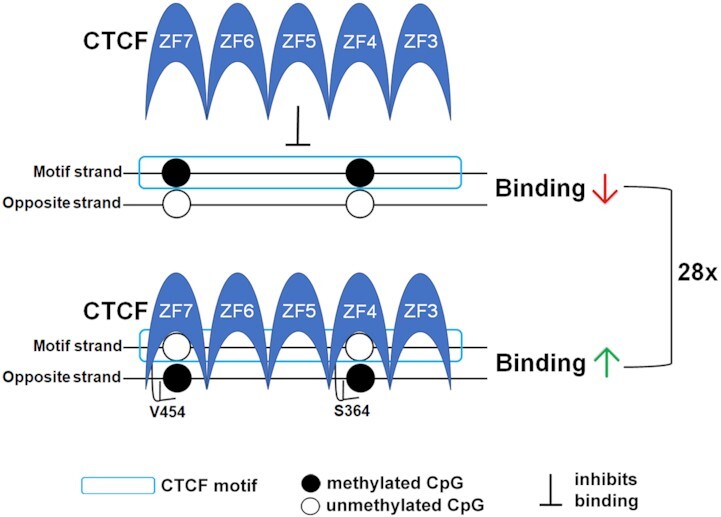
The asymmetry resulting from hemimethylated CpG dyads has opposing effects on CTCF binding. Methylation on the motif strand inhibits binding, whereas methylation on the opposite strand stimulates binding resulting in a 28-fold difference between the two hemimethylation states. The mechanism for sensing methylation on the opposite strand requires valine 454 and serine 364 within CTCF zinc fingers 7 and 4.

## INTRODUCTION

DNA methylation patterns and their faithful inheritance during cell division are essential for appropriate gene expression (1). For vertebrates, cytosines in the CpG palindrome in duplex DNA are generally symmetrically methylated or unmethylated on both strands (2). An exception to this symmetrical methylation occurs when hemimethylated DNA is generated by DNA synthesis during S-phase. This transitory state of inherent asymmetry is generally rapidly converted to symmetrical methylation by DNA methyltransferase 1 which prefers hemimethylated DNA as a substrate (reviewed in (3)). Thus, only low levels of hemimethylation exist in most cells such as somatic mouse tissues (4). On the other hand, maintained hemimethylation of some repetitive sequences has been reported in mouse embryonic stem (ES) cells and in early mouse embryos (4,5). The concept that hemimethylation is simply a transitory state was challenged by the recent discovery of persistent and heritable hemimethylation flanking a subset of CCCTC-binding factor (CTCF) and cohesin binding sites in ES cells, but not in other somatic cell types (6). The fact that the hemimethylation state is heritable after cell division provides a strong suggestion that it might have biological significance.

CTCF has a major role in genomic organization and function (7). Also, binding to its recognition motif sequence is well known to be sensitive to symmetrical DNA methylation, for example in controlling genomic imprinting of the *Igf2/H19* locus (8,9). We therefore used short oligonucleotides (oligos) and recombinant CTCF full-length (CTCF FL) and truncated proteins containing the eleven zinc finger (ZF) domains (CTCF ZF1–11) to investigate potential differential effects of asymmetric DNA methylation states on binding *in vitro*. Methylation on the motif strand inhibited binding; but unexpectedly, methylation on the opposite strand stimulated binding. Potentially, the asymmetry inherent in hemimethylation could be interpreted by CTCF and may serve as a signal for asymmetric cell division in stem cells.

## MATERIALS AND METHODS

### DNA cloning and protein expression

Human CTCF proteins were cloned from the following plasmids: CTCF ZF1–11 (pXC1441) was a gift from Drs Xiaodong Cheng and John Horton (10) and pDONR223_CTCF_WT was a gift from Drs Jesse Boehm, William Hahn, and David Root (Addgene plasmid # 81789; http://n2t.net/addgene:81789;RRID: Addgene_81789) (11). The CTCF ZF1-11 fragment or full-length proteins were cloned into pSUMO vectors for expressing 6xHis-SUMO tagged CTCF proteins. Tagged CTCF proteins were expressed in the Escherichia coli strain BL21-CodonPlus (DE3)-RIL (230245, Agilent). For large-scale (2 L) purification, a colony was inoculated into 100 mL of LB medium containing ampicillin (50 mg/mL) and 25 μM ZnCl_2_ and cultured overnight at 37°C at 170 rpm. Subsequently, the culture was amplified into 2 L of LB medium and was grown for 4–5 h at 28°C to OD_600_ of ∼0.8 and shifted to 16°C. Protein expression was induced with 0.1 mM isopropyl β-D-1-thiogalactopyranoside (IPTG) overnight at 16°C. Cells were harvested by centrifugation at 4000 × g for 20 min at 4°C. Cell pellets were processed immediately or stored at –80°C for future purification.

Cell pellets were resuspended in 150 mL of buffer A (20 mM Tris–HCl (pH 8.0), 25 mM imidazole, 1 M NaCl, 5% (v/v) glycerol, 0.5 mM Tris(2-carboxyethyl)phosphine hydrochloride (TCEP), 25 μM ZnCl_2_) with 1 mM phenylmethanesulfonylfluoride (PMSF). Resuspended cells were lysed in a high-pressure homogenizer (APV) and clarified by centrifugation at 38 700 × g at 4°C for 40 min. The supernatant was collected and loaded onto a 5 ml HisTrap FF column (17525501, Cytiva) pre-equilibrated with buffer A and washed with 10 column volumes (CVs) washing buffer (20 mM Tris–HCl (pH 8.0), 50 mM imidazole, 1 M NaCl, 5% (v/v) glycerol, 0.5 mM TCEP, 25 μM ZnCl_2_). Bound proteins were eluted with elution buffer (20 mM Tris–HCl (pH 8.0), 250 mM imidazole, 1 M NaCl, 5% (v/v) glycerol, 0.5 mM TCEP, 25 μM ZnCl_2_). For 6xHis-SUMO tagged CTCF, pooled protein was subsequently concentrated and loaded onto a Superdex 200 16/600 column (GE28-9893-35, Cytiva) pre-equilibrated with size-exclusion buffer (20 mM Tris–HCl (pH 8.0), 1 M NaCl, 5% (v/v) glycerol, 0.5 mM TCEP, 25 μM ZnCl_2_). The 6xHis-SUMO tagged CTCF protein was eluted at a volume of 80 mL for ZF1-11 and 65 ml for full-length protein. The peak fractions were pooled, concentrated, and fresh-frozen in aliquots after dialysis against a size-exclusion buffer containing 50% glycerol.

For the fluorescence polarization DNA binding assay, both 6xHis-SUMO tagged protein (CTCF ZF1–11) and untagged protein (CTCF ZF1–11 and CTCF full-length) were used, with no change in results based on the presence or absence of the tag. For proteins with the 6xHis-SUMO tag removed, ULP1 Sumo protease was added, and the samples were dialyzed overnight to remove imidazole, then the protein was applied to a 5 ml HisTrap FF column (17525501, Cytiva) pre-equilibrated with buffer A without imidazole. Protein was eluted by a gradient increase in the concentration of imidazole to 250 mM with 10 CVs and subsequently concentrated and loaded onto a Superdex 200 16/600 column pre-equilibrated with size-exclusion buffer. The CTCF protein was eluted at a volume of 88 ml for ZF1–11 and 66 ml for full-length protein. The peak fractions were pooled, concentrated, and fresh-frozen in aliquots after dialysis against a size-exclusion buffer containing 50% glycerol. Protein purity and cleavage completion were analyzed by SDS-PAGE.

### Site-directed mutagenesis

All site-directed mutagenesis was carried out using the QuikChange kit (200521, Agilent) and the constructs were confirmed by DNA sequencing.

### Fluorescence polarization (FP) DNA binding assay

Single-stranded DNA oligos were purchased from IDT and the sequences are shown in Supplementary Table S1. The strand containing the CTCF motif was labeled with 6-carboxy-fluorescein (FAM). Single-stranded oligos were annealed, and the efficiency of the annealing was assessed by running the oligos on a 20% TBE (tris–borate–EDTA) gel. Annealed double-stranded oligos were diluted in DNA binding buffer (20 mM Tris–HCl (pH 7.5), 300 mM NaCl, 5% (v/v) glycerol, and 0.5 mM TCEP) and each oligo (5 nM) was incubated for 15 min at 25°C with a serial dilution of either full-length CTCF or ZF1–11 CTCF protein. The protein concentration ranged from 4000 to 0.244 nM for full-length CTCF and 8000 to 0.488 nM for ZF1-11 CTCF. Duplicate protein serial dilutions were set up for each oligo in a 384-well black assay plate (#3575, Corning) and the assay was repeated giving a total of 4–8 data sets for each oligo-protein pair. The plates were read on a Synergy Neo microplate reader (BioTek). Negative control wells containing only oligo and DNA binding buffer were used as background and subtracted from the polarization values. Polarization (*P*) was then converted to anisotropy (*A*) using the equation (*A*) = (2 × P)/(3 − *P*) (12). Graphpad Prism was used to graph the data and calculate the dissociation constants (K_D_) using the nonlinear regression equation for specific binding with Hill slope. A negative control oligo was included in each assay with a resulting *K*_D_ of ≥900 nM.

### CTCF ZF3–7 and DNA complex modeling

The CTCF ZF3–7 in complex with DNA structure (PDB ID: 5KKQ (10)) was used as the initial structure template. The DNA fragment was manually mutated to Cen-CTCF or H19 in COOT (13), and the protein coordinates and DNA coordinates were saved as receptor and ligand, separately. The docking method MDockPP (14) was run through the provided web server without any constrains. All the other docking parameters were as default. The model was further refined by NPDock DNA–protein complex refinement (15).

### Molecular dynamics simulations

Simulation systems were solvated with TIP3P (transferable intermolecular potential with 3 points) water with 150 mM NaCl, minimized to convergence by steepest descent. 0.1 ns of simulation in the canonical ensemble were then used to relax the solvent and salt with the protein and DNA restrained, and subsequently equilibrated to atmospheric conditions in the isothermal–isobaric ensemble for 5 ns. All simulations used the GROMACS simulation engine (16). Production simulations were performed in the canonical ensemble at 300 K.

### Discovery of hemimethylated CpG dyads within CTCF motifs

Human hemimethylation data from Xu and Corces (2018) (6) and mouse hemimethylation data from Zhao *et al.* (17) were downloaded from GEO database (GSE97394 for Xu and Corces, GSE48229 for Zhao *et al.*). CTCF genomic coordinates for both human GRCh38/hg38 and mouse GRCm38/mm10 were obtained from the R AnnotationHub resource package ‘CTCF 0.99.11’ (18) and intersected with hemimethylated CpG coordinates using Bedtools V2.30 (19). Overlapping CpGs were then filtered for hemimethylation defined as a 50% difference in methylation across a CpG dyad. For the associated genomic regions with nearby genes, gene ontology (GO) analysis was performed using GREAT V4.0.4 (http://great.stanford.edu/public/html/) (20).

## RESULTS AND DISCUSSION

### CTCF binding is regulated by strand-specific CpG methylation

**Figure 1.**
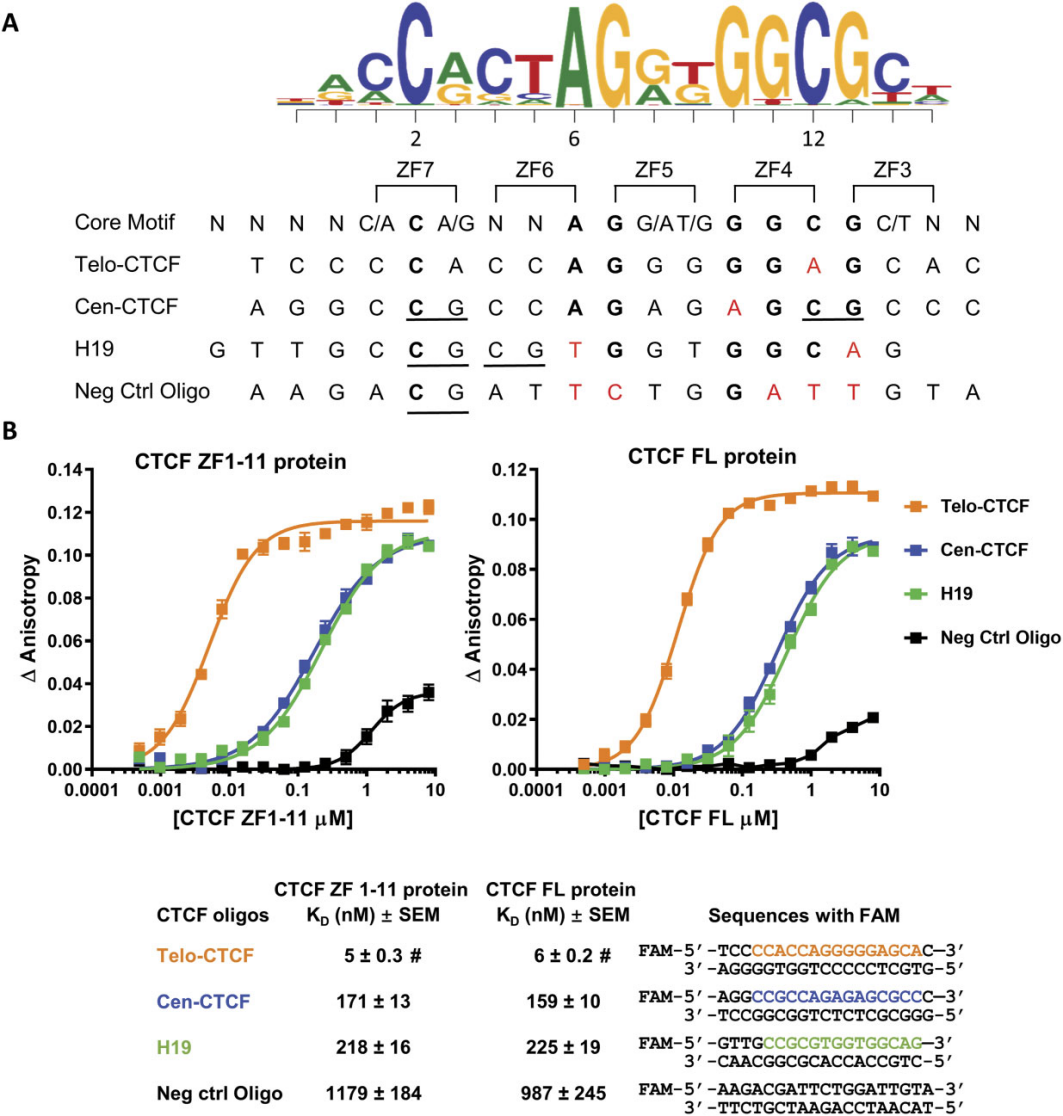
CTCF binds to the Cen-CTCF motif. (**A**) Alignment of the oligo motif strand DNA sequences to the CTCF consensus sequence from *motifbreakR* and to CTCF zinc fingers (ZF) 3–7 as reported by Hashimoto et al. (10). **Bold** = consensus nucleotide, red= mismatch from the consensus sequence, underline = CpG. (**B**) Binding affinity curves and dissociation constants (*K*_D_) of CTCF ZF1–11 and CTCF FL proteins for the oligos defined in (A). Double-stranded oligo sequences are shown with the core motif colored orange for Telo-CTCF, blue for Cen-CTCF, and green for H19. Binding data are represented as mean ± SEM, *n* = 4. **#**: The calculated *K*_D_ of Telo-CTCF is at or near the probe concentration of 5 nM, indicating very tight binding (*K*_D_ ≤ 5–6 nM).

As illustrated in Figure 1A, there are two key cytosines within the CTCF consensus recognition motif at positions 2 and 12. Wang *et al.* reported an enrichment of CpG dinucleotides at these two positions (called position 1 and 11 in their paper) and found that 29% of CTCF motifs contain a CpG at one or both of these positions (21). The C2 and C12 cytosines were shown to interact with the ZF7 and ZF4 domains in the crystal structures reported by Hashimoto et al. (10). To test the effect of hemimethylation of these cytosines on CTCF binding, four CTCF motifs in the form of 18–19 base pair double-stranded oligos (Figure 1) were selected for fluorescence polarization (FP) DNA binding studies (12). We chose to investigate a CTCF site located at the centromeric boundary of a differentially methylated region (DMR) which includes the only known example of a variably imprinted human gene, *nc886/VTRNA2-1* (22,23). We have previously shown that methylation of the *nc886* DMR and the resultant repression of *nc886* is associated with lower body mass index in children and reduced survival in individuals with acute myeloid leukemia (23,24). Given that the nc886 DMR is variably methylated, we hypothesized that the centromeric CTCF site (Cen-CTCF) would be regulated by methylation. The Cen-CTCF binding motif has CpGs at both the C2 and C12 positions and was discovered using *motifbreakR* (https://github.com/Simon-Coetzee/MotifBreakR (25)) to predict whether the rs2346018 A/C polymorphism would affect CTCF binding at this site. The presence of an A provides a consensus nucleotide at position 6 producing a recognition motif for CTCF (Figure 1A). The binding of the Cen-CTCF motif was compared to two positive control motifs. The first is the CTCF motif located telomeric to the *nc886* DMR (Telo-CTCF) which does not contain a CpG site and is frequently occupied by CTCF in multiple cell types based on ENCODE ChIP-seq data. The second positive control is a well-studied binding motif from the *Igf2/H19* imprinting control region (H19) which has previously been shown to interact with CTCF (8–10,26). The H19 motif contains a CpG at position C2 and an adjacent CpG in the triplet interacting with ZF6 (Figure 1A). As a negative control, we used the DNA sequence 5′ to the Cen-CTCF motif which differs considerably from the consensus sequence and is not predicted to be recognized by CTCF.

For the *in vitro* binding experiments, we used both full-length CTCF (CTCF FL) protein and the DNA-binding domain of the protein (CTCF ZF1–11). As shown in Figure 1B, there was strong binding of both CTCF ZF1–11 and CTCF FL proteins (*K*_D_ ∼5 nM) to the positive control, Telo-CTCF oligo. The negative control showed 200-fold less affinity (*K*_D_ ∼1000 nM) establishing the specificity of the assay. The Cen-CTCF and H19 oligos showed intermediate levels of interaction with both CTCF ZF1-11 and CTCF FL proteins with dissociation constants of about 200 nM. Since the results were similar for both the full-length protein and the DNA-binding portion of the protein and only the ZF domains are required for binding to the motif (10,26), further studies to investigate the effects of DNA methylation on CTCF binding were therefore conducted with CTCF ZF1–11 protein using the Cen-CTCF and H19 oligos as ligands.

**Figure 2.**
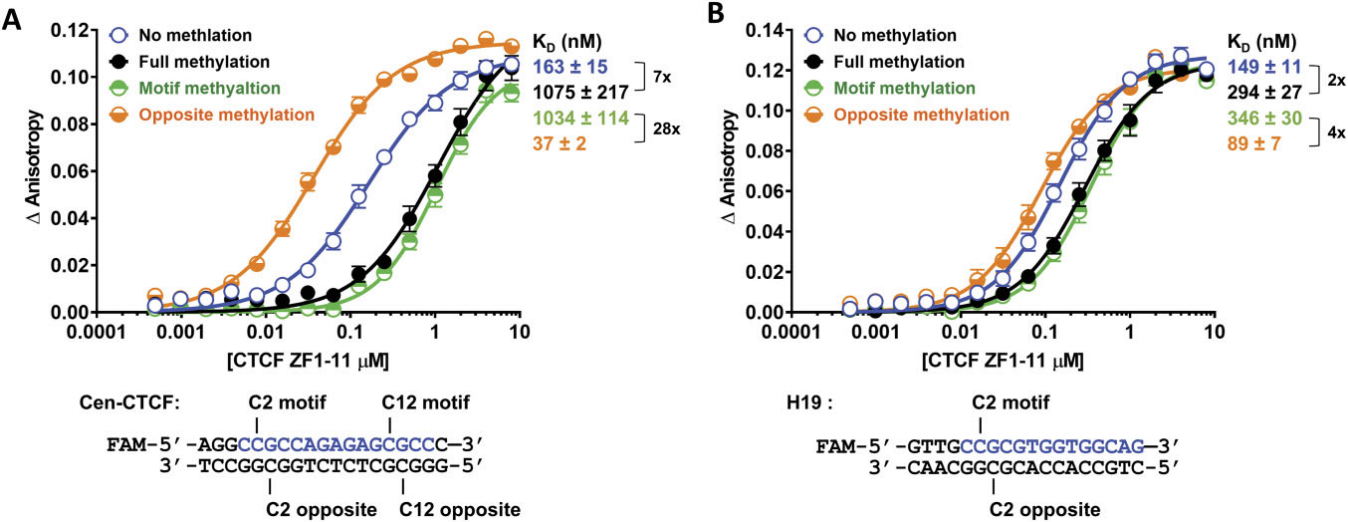
CTCF binding is regulated by strand-specific CpG methylation. (**A** to **B**) Binding affinity curves and dissociation constants (*K*_D_) of CTCF ZF1–11 for the Cen-CTCF (A) and H19 (B) oligos methylated at the indicated positions. Binding data are represented as mean ± SEM, *n* = 4 (Cen-CTCF) and *n* = 8 (H19). Fold change in *K*_D_ between different methylation states is indicated using a bracket. The core motif sequence within the oligo is colored blue.

We next measured the effects of various CpG methylation patterns in the Cen-CTCF and H19 oligos on the binding of CTCF ZF1-11 protein using FP assays (Figure 2). As expected, full methylation of both strands strongly inhibited binding as shown by an increase in the *K*_D_ from 163 to 1075 nM (7-fold difference) (Figure 2A). Methylation of only the motif strand C2 and C12 positions had an almost equivalent effect as full methylation of both strands (*K*_D_ = 1034 nM). On the other hand, methylation of the opposite strand C2 and C12 positions increased binding to a *K*_D_ of 37 nM showing a 28-fold difference in binding between the two hemimethylation states (Figure 2A). Further investigation of the roles of individual C2 and C12 methylation and combinations of methylation showed the dominant role of motif strand C2 methylation in inhibiting binding and the additive nature of opposite strand methylation in stimulating binding (Supplementary Table S2). Given the surprising nature of these observations, we verified the stimulatory effects of hemimethylation using the H19 oligo, since full methylation of this site has been well studied (8,9). As indicated in Figure 1A, this motif has a CpG at the C2 but not the C12 position. Full methylation of the C2 position on both strands or methylation of only the motif strand C2 position changed the *K*_D_ from 149 nM to 294 and 346 nM respectively (Figure 2B). Once again, methylation of the opposite strand stimulated binding (*K*_D_ 89 nM), and the difference between the two hemimethylation states was 4-fold (Figure 2B).

Two previous studies assessed hemimethylation of CTCF binding sites within the imprinting control region upstream of the mouse and human *H19* gene but did not report a change in CTCF binding when the opposite DNA strand was methylated (9,26). One of these studies included the MS1 site from the mouse HS1 insulator (9) which contains the same motif sequence used in the H19 oligo in this paper. The gel mobility shift assay that was used in these previous studies may not have been sensitive enough to observe or quantitate an increase in binding. Here, we find that the effects of strand-specific methylation depend on the actual CTCF target sequence; and the inhibition of binding from motif strand methylation is dominant over the stimulation of binding from opposite strand methylation.

### Hemimethylated CpGs occur within CTCF motifs in embryonic stem cells

Our results that CTCF binding can be regulated by strand-specific hemimethylation states led us to investigate whether any of the hemimethylated CpGs identified in H9 human embryonic stem cells by Xu and Corces (2018) (6) and in undifferentiated E14TG2a mouse embryonic stem cells by Zhao *et al.* (17) are located within a CTCF motif. In the Xu and Corces paper (2018), the authors found that hemimethylated CpGs often flank CTCF motifs and show an asymmetry as to which strand is methylated (6). We used their nascent bisulfite sequencing pulse-chase data to determine whether CpGs within the recognition motif itself are hemimethylated. Using the CTCF consensus sequence data sources as described in Dozmorov *et al.* (18), we identified 102838 CpGs that fall within a CTCF motif of which 803 of these CpGs were found to be hemimethylated across both pulse and chase using a 50% difference in methylation cut-off (Table 1 and Supplementary Table S3). Using the hairpin bisulfite sequencing data from the Zhao *et al.* (2014) paper, we found 189291 CpGs that fall within a CTCF motif of which 2604 are hemimethylated using a 50% difference in methylation cut-off (Table 1 and Supplementary Table S4). It is worth noting that the Cen-CTCF motif is not present in the mouse genome. In the human ES cell line, the Cen-CTCF motif does appear to contain hemimethylation; however, it was not included in our final data set because we limited our results to CpGs with hemimethylation across both pulse and chase and there was missing data at this site. The data from both studies provide evidence that about 1% of CpGs within CTCF motifs are hemimethylated *in vivo*, and there is approximately an even distribution of methylation on either the motif strand or the opposite strand in both mouse and human ES cells (Table 1). This suggests that strand-specific hemimethylation could potentially have opposing effects on CTCF binding *in vivo* as predicted by our *in vitro* data, but further detailed studies in living cells are needed to substantiate this.

**Table 1.** Hemimethylated CpG dyads within CTCF motifs exist in embryonic stem cells

**Data source**	Xu and Corces, 2018 (human)	Zhao *et al.*, 2014 (mouse)
**CpGs located within a CTCF motif**	102 838	189 291
**Hemimethylated CpGs within a CTCF motif**	803* (0.8%)	2604 (1.4%)
**CpGs with hemimethylation on Motif strand**	222	1120
**CpGs with hemimethylation on Opposite strand**	242	1054
**CpGs with hemimethylation on both Motif and Opposite strands**	339	430
**5 most significant GO terms for nearest genes**	Neuron differentiation, Nervous system development, Animal organ development, Cell development, Animal organ morphogenesis	Regulation of granulocyte differentiation, Positive regulation of myeloid cell differentiation, Maintenance of protein location, Positive regulation of myeloid leukocyte differentiation, Cellular response to epidermal growth factor stimulus

Human nascent bisulfite sequencing pulse-chase hemimethylation data from Xu and Corces (2018) and mouse hairpin bisulfite sequencing hemimethylation data from Zhao *et al.* (2014) were downloaded from GEO database (GSE97394 and GSE48229). * Only CpG dyads with hemimethylation across both pulse and chase were included.

To assess the possible biological significance of these hemimethylated CpGs, we used gene ontology (GO) enrichment analysis of the genes which contain or are near the hemimethylated CpGs. We found that hemimethylation at CTCF motifs is enriched within/near genes involved in tissue development and cellular differentiation. The presence of hemimethylated CpGs on the opposite strands may strengthen CTCF binding and provide a mechanism to maintain CTCF at certain critical sites during cell division. Therefore, it is possible that this mechanism could be involved in regulating the differentiation program in stem cells.

### CTCF V454 and S364 sense the presence of methyl groups on the opposite strand

**Figure 3.**
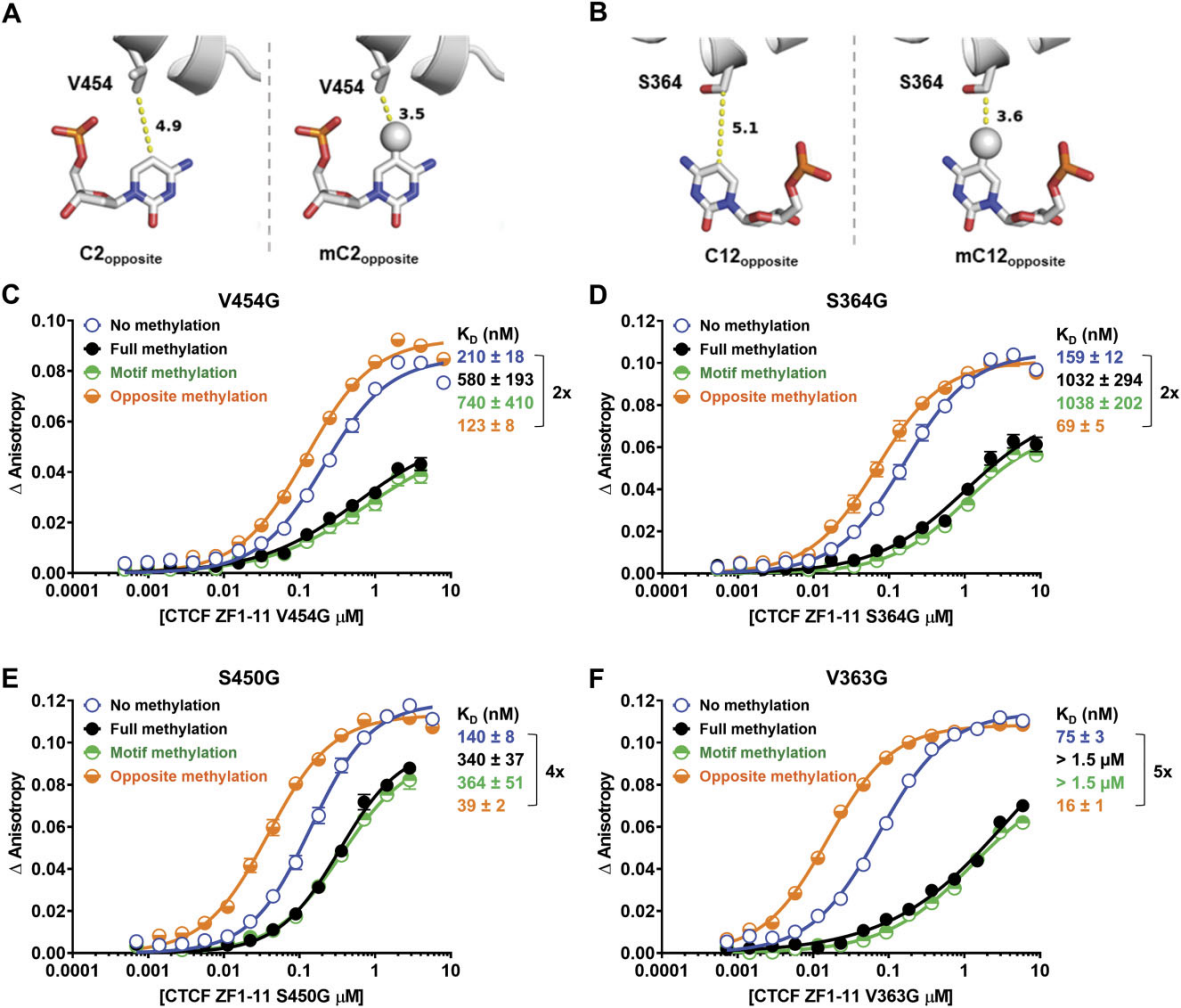
CTCF V454 and S364 sense the presence of methyl groups on the opposite strand. Close-up of V454 (**A**) and S364 (**B**) interactions with opposite strand C2 and C12 positions in the molecular modeling of CTCF ZF3–7 with the unmodified Cen-CTCF motif (left), and opposite stand CpG methylated Cen-CTCF motif (right). Modeling a methyl group onto C2 and C12 opposite positions increases the hydrophobic interaction with V454 of ZF7 and S364 of ZF4. Proteins are in cartoon presentation. Specific nucleotides and key valine and serine residues are in stick presentation. Atoms are colored grey for carbon, blue for nitrogen, brown for phosphorus, and red for oxygen. The 5-methyl groups are decorated with spheres. The numerical numbers indicate the inter-atomic distance in angstroms. (C–F) Binding affinity curves and dissociation constants (*K*_D_) of CTCF ZF1-11 valine-to-glycine and serine-to-glycine mutant proteins for the Cen-CTCF motif methylated at the indicated positions. Mutant residues V454G (**C**) and S450G (**E**) are in close proximity to the C2 position on the opposite strand, and mutant residues S364G (**D**) and V363G (**F**) are in close proximity to the C12 position on the opposite strand. Binding data are represented as mean ± SEM, *n* = 4. Fold change in K_D_ between different methylation states is indicated using a bracket.

To explore the structural basis for the surprising increase in binding of CTCF to methylation on the opposite strand, we utilized the detailed crystallographic data of Hashimoto et al. (PDB ID: 5T00 (10)), which was obtained using a different CTCF motif containing a CA instead of a CpG at the C2 position with the resultant thymine rather than a cytosine on the opposite strand. This opposite strand thymine presents a methyl group in exactly the same geometry as 5-methylcytosine (5mC) and was shown to have hydrophobic interaction with V454 of ZF7 in the existing crystallographic data (PDB ID: 5T00 (10)). In addition, the C beta from the S364 of ZF4 has a hydrophobic interaction with 5mC at the C12 position on the opposite strand. In agreement with this observation, our Molecular Dynamics (MD) simulations using the 5T00 structure and substituting in the Cen-CTCF oligo revealed that V454 and S364 remain in close proximity to the mCpG at positions C2 and C12 on the opposite strand for hundreds of nanoseconds (Supplementary Figure S1). This observation led us to hypothesize that V454 of ZF7 and S364 of ZF4 sense opposite strand methylation through hydrophobic contact with 5mC. Docking of the opposite strands of the C2 and C12 positions of Cen-CTCF was performed *in silico* to investigate the potential interactions between these key cytosines and ZF7 and ZF4 (Figure 3A and B). The Valine residue at position 454 and the Serine residue at position 364 in the protein were found to be in close proximities to the C2 and C12 opposite nucleotides respectively, and therefore may interact with the methyl group of the 5mC through hydrophobic interactions. Molecular modeling predicted that the addition of a methyl group to the C2 opposite position would decrease the interaction distance from 4.9 angstroms (Å) to 3.5 Å and from 5.1 to 3.6 Å for the C12 opposite position (Figure 3A and B). Similar results were found for the C2 position on the H19 motif where 5mC decreased the interaction distance from 4.2 to 3.0 Å suggesting that this mechanism might be general (Supplementary Figure S2).

The likelihood that these predicted interactions with 5mC would increase the binding of CTCF was investigated by exploring the effects of substituting the key residues (V454 and S364) in the protein with glycine (hydrophobic interaction reducing) and investigating the influence of 5mC on binding to Cen-CTCF. As expected, methylation of the motif strand inhibited binding by all mutant proteins (Figure 3C–F). On the other hand, the stimulatory effect of 5mC on the opposite strand was reduced by substitution to glycine at either the V454 or S364 positions (Figure 3C and D), but not at the S450 or V363 positions (Figure 3E and F). These data strongly support the structural predictions by showing that V454 and S364 residues in the CTCF ZFs sense the presence of methyl groups on the opposite strand C2 and C12 positions.

### CTCF binding is regulated by CpG hydroxymethylation

**Figure 4.**
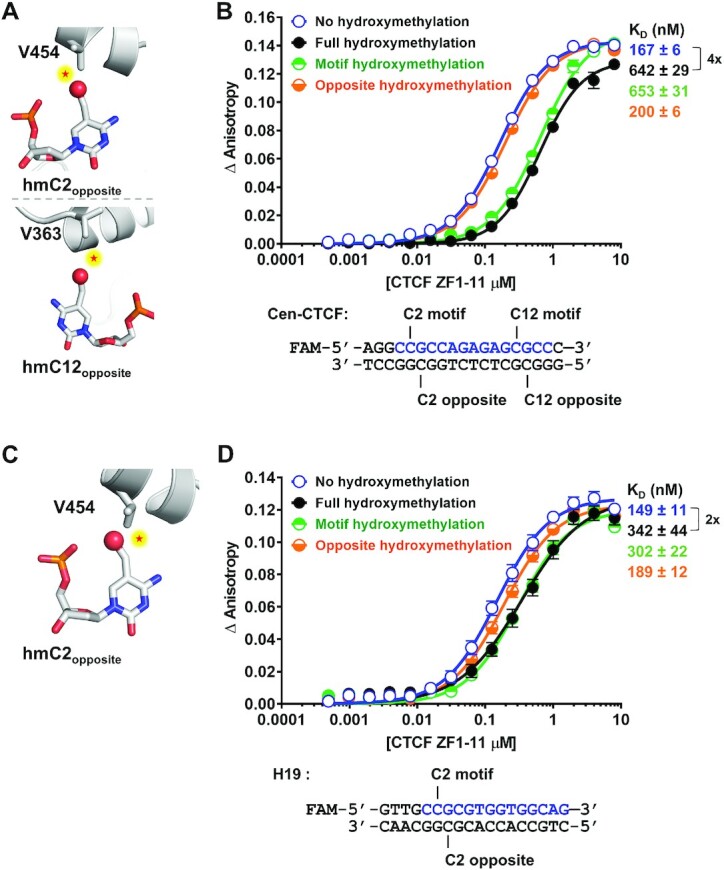
CTCF binding is regulated by CpG hydroxymethylation. (**A**) Modeling of a hydroxymethyl group on the opposite strand C2 and C12 positions of the Cen-CTCF oligo which potentially results in repulsion (indicated by stars) with V454 of ZF7 and S364 of ZF4 in CTCF. (**B**) Binding affinity curves and dissociation constants (*K*_D_) of CTCF ZF1–11 for the Cen-CTCF oligo hydroxymethylated at the indicated positions. (**C**) Modeling of a hydroxymethyl group on the opposite strand C2 position of the H19 oligo potentially results in repulsion (indicated by stars) with V454 of ZF7 in CTCF. (**D**) Binding affinity curves and *K*_D_s of CTCF ZF1–11 for the H19 oligo hydroxymethylated at the indicated positions. Binding data are represented as mean ± SEM, *n* = 4 (Cen-CTCF motif) and *n* = 8 (H19 motif). Fold change in *K*_D_ between different methylation states is indicated using a bracket. The core motif sequence within the oligo is colored blue. For the modeling images, proteins are in cartoon presentation. Specific nucleotides and valines are in stick presentation. Atoms are colored grey for carbon, blue for nitrogen, brown for phosphorus, red for oxygen. The hydroxymethyl groups are decorated with red spheres.

Several studies have reported changes in 5-hydroxymethylcytosine (5hmC) levels at CTCF binding sites (27–30); however, little is known about the effects of 5hmC on CTCF binding. The 5hmC base is predicted to not only introduce a hydrophilic group, but also to sterically obstruct the interactions between the CTCF V454 and S364 with the opposite strand C2 and C12 positions of Cen-CTCF and C2 of H19 (Figure 4A and C). Not shown, is the prediction that 5hmC on the motif strand would still inhibit binding given that it is larger than 5mC. We tested these predictions using the Cen-CTCF and H19 oligos containing 5hmC at the target positions (Figure 4B and D). Full hydroxymethylation of the CpGs within the Cen-CTCF oligo or hydroxymethylation on the motif strand resulted in a 4-fold reduction in the binding affinity (Figure 4B), confirming that 5mC or 5hmC at the C2 position sterically obstructs D451 in CTCF (10). The presence of 5hmC on the opposite strand had no effect or subtle effects on the binding of CTCF (Figure 4B). For the H19 oligo, hydroxymethylation on both strands of the oligo or only on the motif strand resulted in a 2-fold reduction in the binding affinity (Figure 4D). As with the Cen-CTCF oligo, hydroxymethylation of the opposite strand of the H19 oligo had only a subtle effect with slightly diminished binding affinity relative to the unmethylated oligo (Figure 4D). Therefore, the substitution of 5hmC for 5mC on the opposite strand mitigated the stimulation, whereas it maintained the inhibitory effects on the motif strand, albeit to a lesser extent than 5mC. Our finding that hydroxymethylation can inhibit the binding of CTCF to DNA is in line with a study by Marina *et al.* (2016) in which they show by electrophoretic mobility shift assay that DNA probes from the *CD45* and *KCNA2B* genes with the modifications 5mC, 5hmC, and 5fC have minimal interaction with CTCF (31). Variable hydroxymethylation might therefore add an additional level of control to CTCF binding. Previous reports have shown that hydroxymethylation is increased at weak and variable CTCF sites (29) and have suggested a role for hydroxymethylation in maintaining a poised chromatin state (28,29). Thus, hydroxymethylation may represent a dynamic intermediate that maintains the motif in a poised state ready for changes in cytosine modifications that could stimulate the binding of CTCF.

## CONCLUSIONS AND IMPORTANCE

The recent discovery of heritable hemimethylation states in ES cells (6) has challenged the long-held assumption that hemimethylation is simply an intermediate to full symmetrical methylation and therefore of little biological significance. Our results that different hemimethylation states at CTCF binding sites can change binding affinity by as much as 28-fold suggests that there may indeed be a function for hemimethylation in the organization of the genome, particularly in ES cells. We were helped considerably in defining the mechanism for these effects by the detailed structures published by Hashimoto *et al.* (10) and were able to confirm the mechanism by mutating the relevant zinc fingers in CTCF. Our results are particularly intriguing because hemimethylation at a single CTCF motif could potentially cause large-scale alterations of the epigenome in ES cells. A recent study by Gabriele *et al.* demonstrated that even CTCF topologically associated domain boundaries, which are thought to be stable interactions, can be highly dynamic and transient in nature (32). The regulation of CTCF sites by specific CpG modified states in a strand-specific manner, like hemimethylation and hydroxymethylation, may provide a mechanism to explain the transient and dynamic nature of CTCF-mediated chromatin interactions.

The phenomenon of asymmetric cell division was identified by Edwin Conklin >100 years ago (33) and many mechanisms likely participate in the generation of cellular diversity (34). While there is no evidence yet that DNA methylation plays a role in this process, hemimethylation may provide a mechanism for asymmetric cell division given that it generates two sequence-identical DNA duplexes with strongly differing potentials to bind CTCF. In this regard, it is interesting that hemimethylation is much less common in the differentiated progeny of ES cells (6) suggesting that resolution of hemimethylation may be associated with cell differentiation.

## DATA AVAILABILITY

All data are available from the corresponding author upon request.

## Supplementary Material

gkad293_Supplemental_Files
