# Supplementary material for: DNA strand asymmetry generated by CpG hemimethylation has opposing effects on CTCF binding
Source: Nucleic Acids Res. 2023 Apr 24;51(12):5997–6005. doi: 10.1093/nar/gkad293 (PMC10325916; doi:10.1093/nar/gkad293)
Supplement: gkad293_Supplemental_Files [file gkad293_supplemental_files.zip › Thomas et al NAR Supplementary Data 2023.pdf]

SUPPLEMENTARY DATA

DNA strand asymmetry generated by CpG hemimethylation has opposing effects on CTCF binding

Stacey L. Thomas[1, †], Ting-Hai Xu[1, 2, †], Brittany L. Carpenter[1], Steven E. Pierce[3], Bradley M. Dickson[1], Minmin Liu[1], Gangning Liang[4], and Peter A. Jones[1, *]

[1] Department of Epigenetics, Van Andel Institute, Grand Rapids, MI, 49503, USA
[2] Department of Structural Biology, Van Andel Institute, Grand Rapids, MI, 49503, USA
[3] Department of Neurodegenerative Science, Van Andel Institute, Grand Rapids, MI, 49503, USA
[4] Department of Urology, Keck School of Medicine, University of Southern California, Los Angeles, CA, 90089, USA

* To whom correspondence should be addressed. Tel: 616-234-5041; Email: peter.jones@vai.org

[†]The authors wish it to be known that, in their opinion, the first 2 authors should be regarded as joint First Authors.

This PDF file includes:

Tables S1 to S2
Figures S1 to S2

Table S1. Oligonucleotide sequences used for the FP DNA binding assay.

Oligo name	Sequence
Telo-CTCF	FAM-5′-TCC CCA CCA GGG GGA GCA C-3′<br>3′-AGG GGT GGT CCC CCT CGT G-5′
H19	FAM-5′-G TTG CCG CGT GGT GGC AG-3′<br>3′-C AAC GGC GCA CCA CCG TC-5′
H19 full methylation	FAM-5′-G TTG C**M**G CGT GGT GGC AG-3′<br>3′-C AAC GG**M** GCA CCA CCG TC-5′
H19 motif methylation	FAM-5′-G TTG C**M**G CGT GGT GGC AG-3′<br>3′-C AAC GGC GCA CCA CCG TC-5′
H19 opposite methylation	FAM-5′-G TTG CCG CGT GGT GGC AG-3′<br>3′-C AAC GG**M** GCA CCA CCG TC-5′
H19 full hydroxymethylation	FAM-5′-G TTG C**H**G CGT GGT GGC AG-3′<br>3′-C AAC GG**H** GCA CCA CCG TC-5′
H19 motif hydroxymethylation	FAM-5′-G TTG C**H**G CGT GGT GGC AG-3′<br>3′-C AAC GGC GCA CCA CCG TC-5′
H19 opposite hydroxymethylation	FAM-5′-G TTG CCG CGT GGT GGC AG-3′<br>3′-C AAC GG**H** GCA CCA CCG TC-5′
Cen-CTCF	FAM-5′-AGG CCG CCA GAG AGC GCC C-3′<br>3′-TCC GGC GGT CTC TCG CGG G-5′
Cen-CTCF full methylation	FAM-5′-AGG C**M**G CCA GAG AG**M** GCC C-3′<br>3′-TCC GG**M** GGT CTC TCG **M**GG G-5′
Cen-CTCF motif C2 methylation	FAM-5′-AGG C**M**G CCA GAG AGC GCC C-3′<br>3′-TCC GGC GGT CTC TCG CGG G-5′
Cen-CTCF motif C12 methylation	FAM-5′-AGG CCG CCA GAG AG**M** GCC C-3′<br>3′-TCC GGC GGT CTC TCG CGG G-5′
Cen-CTCF motif methylation	FAM-5′-AGG C**M**G CCA GAG AG**M** GCC C-3′<br>3′-TCC GGC GGT CTC TCG CGG G-5′
Cen-CTCF opposite C2 methylation	FAM-5′-AGG CCG CCA GAG AGC GCC C-3′<br>3′-TCC GG**M** GGT CTC TCG CGG G-5′
Cen-CTCF opposite C12 methylation	FAM-5′-AGG CCG CCA GAG AGC GCC C-3′<br>3′-TCC GGC GGT CTC TCG **M**GG G-5′
Cen-CTCF opposite methylation	FAM-5′-AGG CCG CCA GAG AGC GCC C-3′<br>3′-TCC GG**M** GGT CTC TCG **M**GG G-5′
Cen-CTCF full hydroxymethylation	FAM-5′-AGG C**H**G CCA GAG AG**H** GCC C-3′<br>3′-TCC GG**H** GGT CTC TCG **H**GG G-5′
Cen-CTCF motif hydroxymethylation	FAM-5′-AGG C**H**G CCA GAG AG**H** GCC C-3′<br>3′-TCC GGC GGT CTC TCG CGG G-5′
Cen-CTCF opposite hydroxymethylation	FAM-5′-AGG CCG CCA GAG AGC GCC C-3′<br>3′-TCC GG**H** GGT CTC TCG **H**GG G-5′
Neg Ctrl Oligo	FAM-5′- AAG ACG ATT CTG GAT TGT A-3′<br>3′- TTC TGC TAA GAC CTA ACA T-5′

The strand containing the CTCF motif was labeled with FAM. Modifications at the indicated positions. M: 5-methylcytosine, H: 5-hydroxymethylcytosine.

Table S2. Effect of CpG methylation on the binding of CTCF to the Cen-CTCF oligo.

Cen-CTCF Oligo	K_D (nM) ± SEM	K_D Fold Change
No methylation	163 ± 15	
Full methylation	1075 ± 217	− 7
Motif C2 +C12	1034 ± 114	− 6
Motif C2	949 ± 165	− 6
Motif C12	428 ± 37	− 3
Opposite C2 + C12	37 ± 2	+ 4
Opposite C2	81 ± 5	+ 2
Opposite C12	77 ± 5	+ 2
Motif C2 + Opposite C2	799 ± 110	− 5
Motif C2 + Opposite C12	839 ± 74	− 5
Motif C12 + Opposite C2	243 ± 18	− 2
Motif C12 + Opposite C12	303 ± 28	− 2

Dissociation constants (K_D) of CTCF ZF1-11 for the Cen-CTCF oligo methylated at the indicated positions. Binding data are represented as mean ± SEM, n = 4. K_D Fold Change is relative to the unmethylated oligo (No methylation).

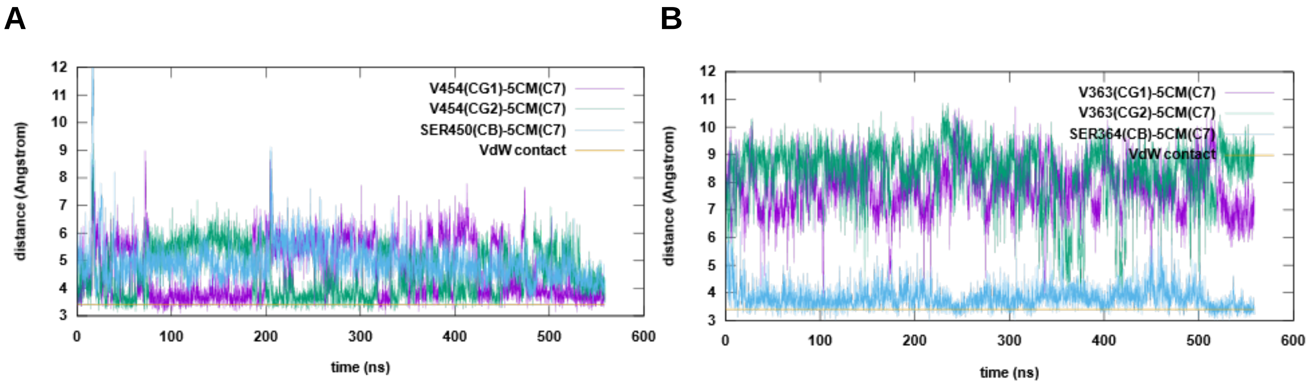

Figure S1. Molecular Dynamics simulations using the 5T00 structure (Hashimoto et al., 2017) and substituting in the Cen-CTCF oligo. (**A**) Proximity of V454 and S450 to the mCpG at position C2 on the opposite strand. (**B**) Proximity of V363 and S364 to the mCpG at position C12 on the opposite strand.

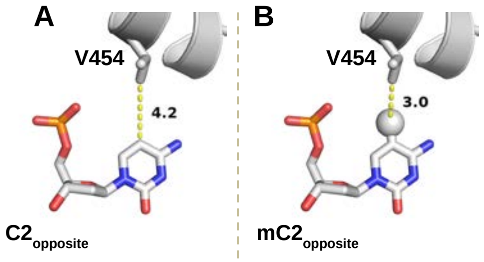

Figure S2. Modeling of CTCF ZF3-7 with the H19 oligo. (**A**) Close-up of V454 interactions with the opposite strand C2 position of the unmodified H19 motif. (**B**) Modeling a methyl group onto the opposite strand C2 position increases the hydrophobic interaction with V454 of ZF7. Proteins are in cartoon presentation. Specific nucleotides and key valines are in stick presentation. Atoms are colored grey for carbon, blue for nitrogen, brown for phosphorus, and red for oxygen. The 5-methyl group is decorated with a sphere. The numerical numbers indicate the potential inter-atomic distance in angstroms.
